# Chronic *Cystoisospora belli* infection in an immunocompetent Myanmar refugee – microscopy is not sensitive enough

**DOI:** 10.1186/s12879-016-1558-3

**Published:** 2016-05-21

**Authors:** Sze-Ann Woon, Rongchang Yang, Una Ryan, Peter Boan, David Prentice

**Affiliations:** Department of Microbiology and Infectious Disease, PathWest Laboratory Medicine WA and Royal Perth Hospital, Perth, Australia; School of Veterinary and Life Sciences, Murdoch University, Perth, Australia; Department of Internal Medicine, Royal Perth Hospital, Perth, Australia; Department of Infectious Disease, National University Hospital, 5 Lower Kent Ridge Road, Singapore, 119074 Singapore

## Abstract

**Background:**

Cystoisosporiasis is an opportunistic infection seen more commonly in patients with acquired immunodeficiency syndrome. Although uncommon, *Cystoisospora* infection can occur in immunocompetent individuals but tend to be benign and self-limiting. Chronic infection however, has been described but diagnosis can often be challenging and requires a high clinical index of suspicion.

**Case presentation:**

We present a case of delayed diagnosis of *Cystoisospora belli* (*C. belli*) in an immunocompetent 28-year-old refugee from Myanmar. She had a history of chronic diarrhea where exhaustive investigations over many years failed to reveal a diagnosis. *Cystoisospora belli* cysts were finally detected in stool 4 years after investigation commenced, and PCR testing on stored colon biopsies amplified a molecular product with 99 % sequence homology to *C. belli*. The patient improved promptly with trimethoprim-sulfamethoxazole treatment.

**Conclusion:**

In the appropriate clinical context we suggest molecular testing for *C. belli* or an empirical therapeutic trial.

## Background

*C. belli* is an obligate intracellular coccidian parasite and is a recognized agent of enteric disease. It has a global distribution with predominance in the tropics and subtropics [1]. Intestinal parasitic infection is common in Myanmar, as shown in a screening study of Myanmar migrant workers, demonstrating intestinal parasites in 62.3 % of migrants [2]. However, there is no data in the literature regarding rates *of C. belli* in Myanmar. *Cystoisospora* infection is characterized by watery diarrhea, sometimes with fever, abdominal pain, nausea and vomiting. Peripheral eosinophilia is commonly noted as distinct from other coccidian parasites. Immunosuppressed patients present with more severe diarrhea and can have extra-intestinal manifestations whereas infection in immunocompetent patients tends to be self-limiting. Chronic infection affecting immunocompetent individuals can occur but can be challenging to diagnose owing to the intermittent shedding of the oocysts and low sensitivity of standard microscopic techniques in detecting the cysts. This can potentially lead to delayed diagnosis and treatment as in the case of our patient where the diagnosis was eventually made after 4 years of extensive and repeated investigations by PCR testing of colonic biopsies.

### Clinical record

A 28-year-old female from Myanmar was referred for investigation of diarrhea in 2010, shortly after arrival as a refugee to Perth, Western Australia. Diarrhea up to 10 times per day had been ongoing since age 8, occasionally associated with nausea, vomiting or abdominal pain but without steatorrhea, blood in the stool or fevers. Low body mass index of 16 kg/m^2^ was persistent despite attempts to gain weight. Hospital admission several times per year was required due to volume depletion and hypokalemia.

Differential white cell count was significant for persistent eosinophilia, usually 1-2 x 10^9^ cells/L (N 0.00-0.50 x 10^9^ cells/L), with a peak level of 6.7 x 10^9^ cells/L. IgE was also markedly elevated (4100 KU/L, N <210 KU/L]). MRI of the pancreas and biliary tree, CT abdomen, CT enteroclysis, autoimmune screen, coeliac antibodies, several upper GI endoscopies and colonscopies were all unremarkable. Serology for HIV, *Schistosoma* and *Strongyloides* infections was negative. Histopathology of endoscopic biopsy samples demonstrated mild duodenitis and colitis characterized by increased numbers of plasma cells and lymphocytes. Granulomas were absent, and there were normal numbers of eosinophils. Parasites were not detected in 10 stool samples from the years 2010–2012, specifically examined for parasites including *Cryptosporidium*, *Cyclospora* and *Cystoisospora* by centrifugation (500 x g for 10 min), concentrated wet mount and modified safranin stain. *Microsporidia* were not detected in 3 stools examined by modified trichrome stain. Charcot-Leyden crystals were noted in a number of stool samples. Diarrhea persisted despite empirical treatment of intestinal parasites with metronidazole (400 mg orally, 8-hourly for 5 days) and albendazole (400 mg orally, 12-hourly for 3 days).

**Fig. 1:**
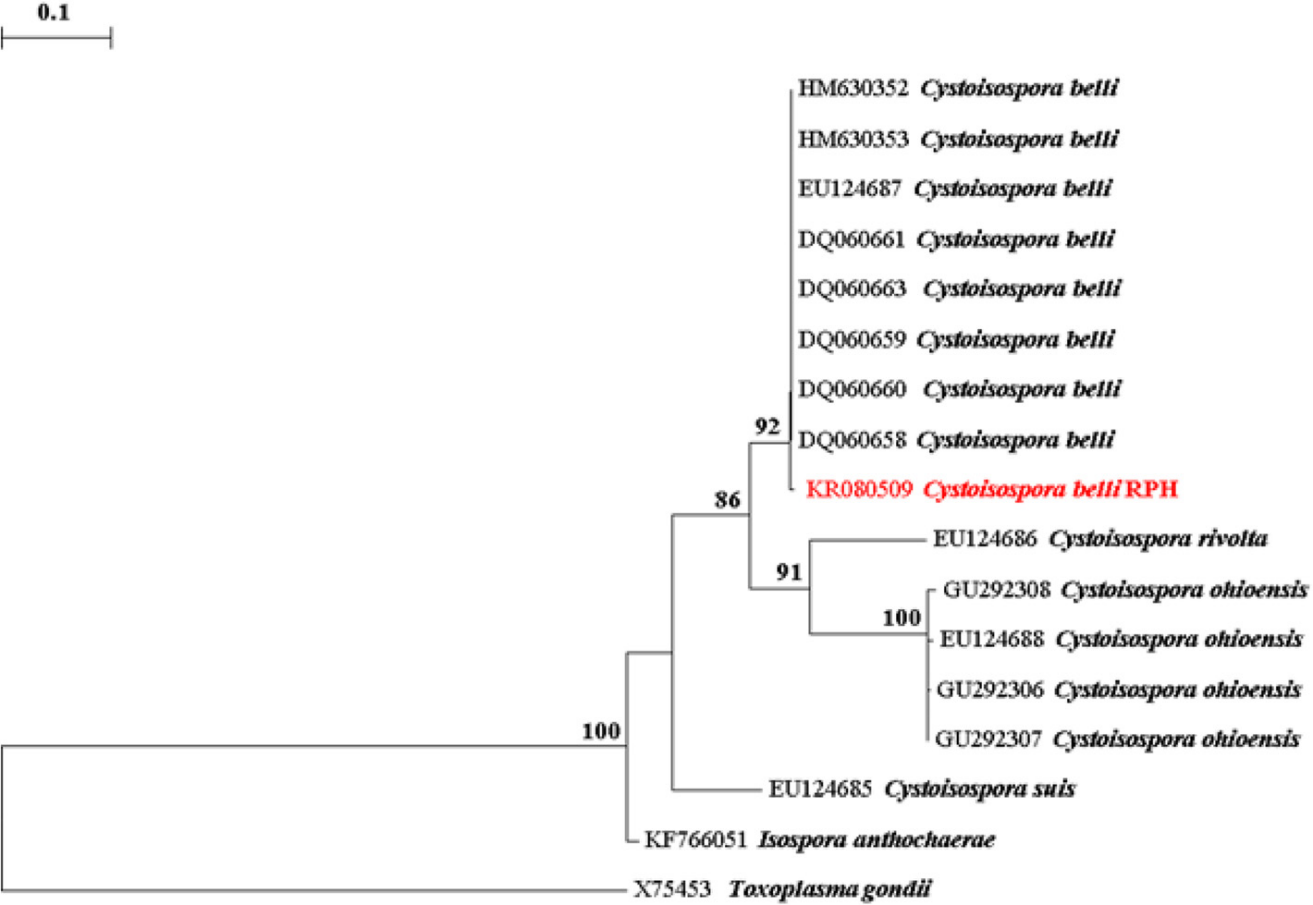
Evolutionary relationships of *Cystoisospora belli* inferred by distance analysis of ITS rRNA sequences. Percentage support (>70 %) from 1000 pseudoreplicates is indicated at the left of the supported node

**Fig. 2:**
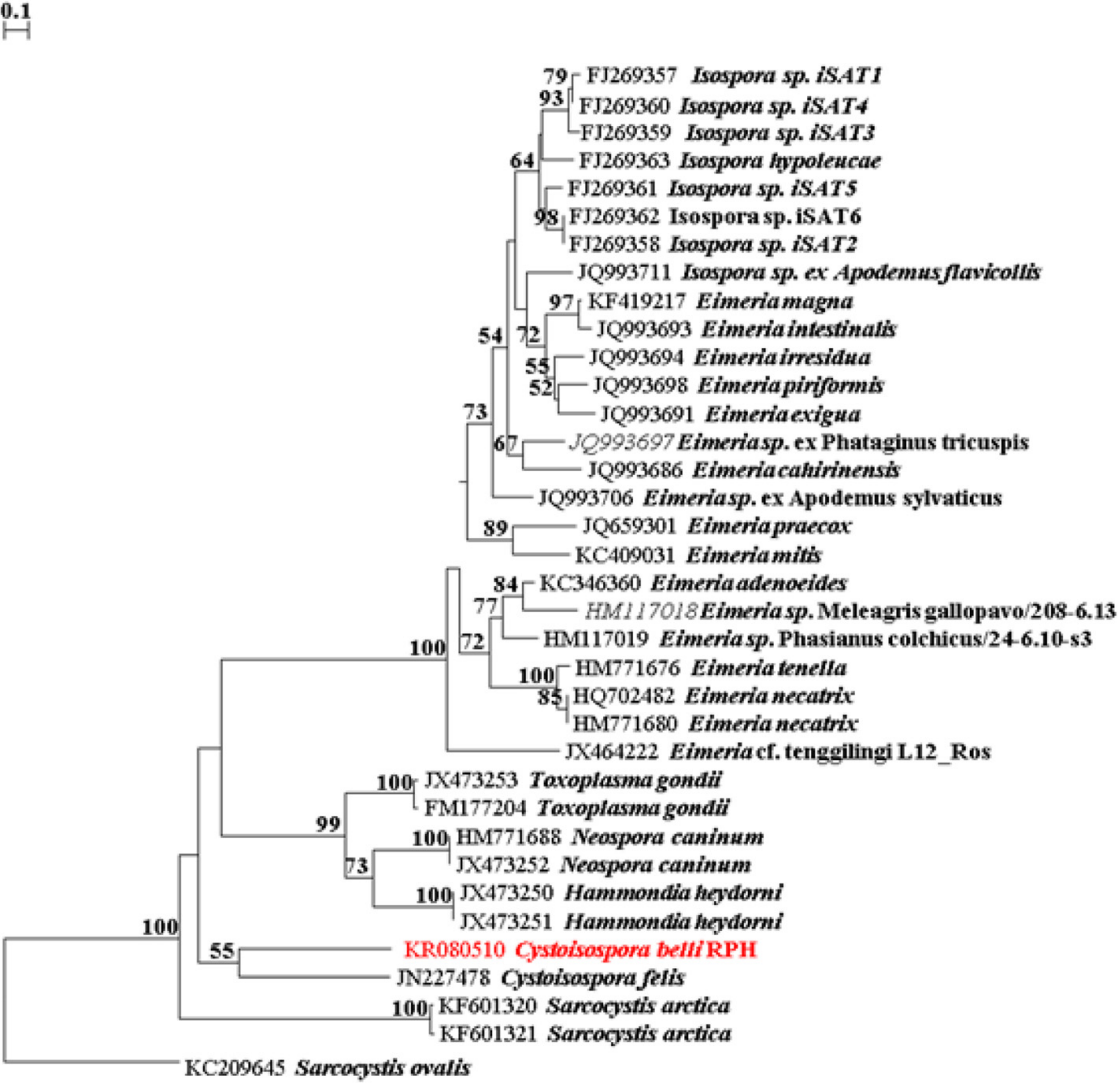
Evolutionary relationships of *Cystoisospora belli* inferred by distance analysis of COI sequences. Percentage support (>70 %) from 1000 pseudoreplicates is indicated at the left of the supported node

Finally *C. belli* oocysts were detected in a stool sample in 2014. Stool not being available for molecular testing, we then tested by PCR stored paraffin sections of colon biopsies obtained at colonoscopy in 2012, detecting *C. belli* in 3 of 5 biopsies. We deparaffinised with xylene, and extracted with digestion buffer (10 mmol/L Tris-HCl, 50 mmol/L KCl, 1.5 mmol/L MgCl2, 0.5 % Tween 20) plus proteinase K 20 mg/mL. Amplification of a 404 bp region of the *Cystoisospora* ribosomal RNA (rRNA) internal transcribed spacer (ITS) locus by nested PCR from the 5 DNA samples was conducted as described by Johnson et al. [3] The 5 samples were also amplified at the mitochondrial cytochrome oxidase gene (COI) locus by a nested PCR as described by Dolnik et al. [4] and Yang et al. [5] Secondary PCR products were gel purified using an in house filter tip method without any further purification for downstream sequencing as previously described [6]. The PCR amplicons were sequenced with the internal ITS and COI PCR primers respectively, in both directions using a BigDye® Terminator Kit (v3.1) (Life Technologies, Foster City, California). The results of the sequencing reactions were analysed and edited using Finch *TV*® v 1.4.0 [7]. and compared to existing *Cystoisospora* spp. ITS and COI sequences on GenBank using BLAST searches and aligned with reference species from GenBank using Clustal W [8]. Phylogenetic analyses were conducted for *Cystoisospora* spp. at the ITS and COI loci with additional isolates from GenBank. Distance estimation was conducted using TREECON [9], based on evolutionary distances calculated with the Tamura-Nei model and grouped using Neighbour-Joining. Maximum Likelihood (ML) analyses were conducted using MEGA version 6 (MEGA6: Molecular Evolutionary Genetics Analysis software, Arizona State University, Tempe, Arizona, USA). Bootstrap analyses were conducted using 1000 replicates to assess the reliability of inferred tree topologies. Three of the five samples were positive by PCR at the ITS locus. Of these three, there was sufficient DNA from two of these positives for sequencing analysis. Both samples produced 100 % identical 414 bp sequences that were 99.5 % identical to *C. belli* from other human-derived *Cystoisospora* isolates available from GenBank (accession numbers: MH630353, EU124687, HM630352 and DQ065658 -DQ065663) (Fig. 1-Distance tree shown). At the COI locus, two of the five samples were positive by PCR. Sequences were obtained from both 223 bp amplicons. Unfortunately, no *C. belli* COI sequences were available in GenBank and therefore the sequences obtained in the present study exhibited 85.5 % similarity to *C. felis* (GenBank accession number: JN473253) (Fig. 2-Distance tree shown). This is the first report of sequence data for *C. belli* at the COI locus. Distance and ML analysis produced identical tree topologies (data not shown).

There was transient resolution of diarrhea with trimethoprim-sulfamethoxazole (TMP-SMX) one double strength tablet (160 mg TMP/800 mg SMX) twice daily for 10 days, with complete resolution of symptoms with TMP-SMX retreatment for one month at the same daily dose. Microscopic examinations of 12 subsequent stool samples were negative with normalization of eosinophilia and no recurrence of diarrhea.

## Discussion

Chronic and recurrent infection in immunocompetent patients such as occurred in our patient have been previously described [10]. Jongwutiwes et al. [11] report a 54 year old Thai man with a 10 year history of intermittent diarrhea. Several initial stools were negative for parasites by microscopy, with *C. belli* oocysts detected in stool and jejunal biopsy 3 years after initial symptoms. He responded promptly to TMP-SMX however needed several courses before diarrhea was cured. Kim et al. [12] report a 2 year history of diarrhea and 35 kg weight loss in a 70 year old alcoholic Korean man. There was prominent peripheral eosinophilia, and stool examination on 3 occasions did not detect parasites. The diagnosis of chronic *C. belli* infection was finally made by demonstration of a number of the parasite stages in jejunal biopsy. The patient was successfully treated with 1 month of TMP-SMX.

Diagnosis of *Cystoisospora* infection can be challenging owing to the intermittent and low grade shedding of oocysts, as demonstrated in our case and also the other reported cases of chronic infection. The sensitivity of any microscopic technique is unknown without an optimal gold standard for diagnosis. In a study comparing stains for the detection *C. belli*, auto-fluorescence and auramine-O staining had 100 % sensitivity and specificity compared to the modified Zeihl-Neelsen stain, while wet mount with iodine staining showed 54 % sensitivity [13]. Similarly, another study showed auto-fluorescent staining detected *C. belli* in twice as many stools as the iodine stain [14]. Repeated sampling and examination by trained microscopists using specific methods is suggested [7], however molecular testing [15] may be more sensitive as has been demonstrated for *Cryptosporidium* PCR compared to microscopy [16, 17]. In evaluating a multiplex PCR to detect *Cystoisospora*, *Cyclospora* and *Microsporidia* in stool, Taniuchi et al demonstrated 93 % sensitivity of the *Cystoisospora* PCR assay compared to microscopy (26 PCR positive of 28 microscopy positive). 14 of the total 208 microscopy negative samples tested positive by PCR. Some of the available discrepant samples tested positive by an alternative singleplex real-time PCR *C. belli* assay, suggesting higher sensitivity of molecular techniques compared to microscopy [18]. A separate study of a real-time PCR assay found it to be at least as sensitive (100 % sensitive, 21 samples) as microscopy for the detection of *C. belli. C. belli* PCR was negative on all 147 negative controls [19]. Murphy et al. [20] report a 44 year old HIV infected Mexican with diarrhea and weight loss. Stool microscopy and ileal biopsy did not reveal an infectious agent, but PCR of the ileal biopsy material using universal primers (ITS1, ITS2, 28S rRNA genes) detected an ITS1 product, which by molecular sequencing had good homology with reference sequences of *C. belli*. In the present study, nested PCR and sequence analysis at two loci was used to detect and confirm *C. belli* in three of five paraffin-embedded colon biopsy specimens.

## Conclusion

We present the case of a refugee with chronic diarrhea, Charcot-Leyden crystals in the stool, and peripheral eosinophilia. Chronic intestinal parasitic infection was suspected but could not be proven until *C. belli* was detected on the 11^th^ stool examination. We deduced that *C. belli* was the cause of chronic diarrhea due to high epidemiological risk, persistent eosinophilia, positive molecular testing on stored biopsies, response to TMP-SMX, and absence of an alternative diagnosis despite exhaustive investigation. We conclude that standard microscopic methods to detect *C. belli* were not sensitive enough to detect the pathogen. Potentially there are a large number of undiagnosed cases worldwide, where molecular testing should be pursued or empirical treatment considered.

## Ethics approval and consent to participate

Not applicable.

## Consent for publication

Written informed consent was obtained from the patient for publication of this case report. A copy of the written consent is available for review by the Editor of this journal.

## Availability of data and materials

Phylogenetic material deposited in Genbank (GenBank accession numbers: KR0805009 for ITS sequences and KR0805010 for COI sequences) and TreeBase (ID 19089).

## Abbreviations

CT: computed tomography
DNA: deoxyribonucleic acid
GI: gastrointestinal
HIV: human immunodeficiency virus
IgE: immunoglobulin E
MRI: magnetic resonance imaging
PCR: polymerase chain reaction
RNA: ribonucleic acid
